# Eosinophilic Fasciitis Presenting as Chronic Nonspecific Symptoms in a Young Adult

**DOI:** 10.1093/omcr/omad131

**Published:** 2023-12-19

**Authors:** Mansour Khaleel, Mohammed Ayyad, Nabil C N Khalil, Omar Tarayrah, Muath Tumezeh, Sami Bannoura

**Affiliations:** Department of Internal Medicine, Makassed Hospital, Jerusalem, IL; Internal Medicine Department, Al Quds University, Abu Dis, State of Palestine; Department of Internal Medicine, Makassed Hospital, Jerusalem, IL; Department of Internal Medicine, Makassed Hospital, Jerusalem, IL; Department of Internal Medicine, Makassed Hospital, Jerusalem, IL; Department of Pathology, Makassed Hospital, Jerusalem, IL

## Abstract

Eosinophilic fasciitis (EF) is a rare inflammatory disease affecting various connective tissues. It is characterized by induration of the skin as well as scleroderma-like manifestations that are progressive and bilaterally symmetrical. Additionally, the joints and muscles are commonly involved, and rarely, there can be involvement of internal organs. The diagnosis of EF is based on clinical, laboratory, radiological, and a full-thickness skin biopsy involving the fascia. The biopsy is often diagnostic and shows eosinophilic inflammation. Systemic corticosteroids specifically prednisone and prednisolone remain the preferred treatment of choice and may be combined with immunosuppressive therapy in a subset of patients. We herein report a patient complaining of chronic nonspecific symptoms that were diagnosed with EF. The patient was treated with steroids with marked improvement of his overall condition.

## INTRODUCTION

Eosinophilic fasciitis (EF) is a rare connective tissue disease mainly affecting the skin and soft tissue. The disorder is characterized by eosinophilic inflammation of the deep fascia leading to symmetrical and painful swelling of the upper and lower extremities, with concurrent progressive induration and thickening of the skin [1]. Interestingly, EF occurs most commonly in middle-aged individuals, with a peak incidence between 40 and 50 years of age [1]. Although multiple theories have emerged aiming to understand the underlying cause of EF, the etiology of this condition remains unknown. Conversely, the pathogenesis of this disorder is considered to include a combination of an immuno-inflammatory process along with an allergic component [2]. The definitive diagnosis is established with a full-thickness skin biopsy involving the fascia, which includes subcutaneous cellular tissue and muscle fascia [3]. Due to the rarity of this disease, there are currently no definitive therapeutic guidelines. However, prednisone and other corticosteroids have demonstrated efficacy and remain to be the standard therapy for EF, although some patients have been shown to improve spontaneously without any therapeutic intervention [2].

## CASE REPORT

A 30-year-old male presented for evaluation of bilateral upper and lower extremities swelling. The patient also reported generalized fatigue, morning stiffness, and erythema of his fingers over the past two months. He also had night sweats and subjective fevers. Over the past five months, there has been an unexplained weight loss of 18 kilograms, verified through changes in clothing fit and the readings on a scale. The patient also reported having multiple episodes of frequent diurnal urination, as well as nocturnal urinary incontinence waking him up to five times per night.

On physical examination, there was generalized tightening of the skin and underlying soft tissue thickening in the distal upper and lower extremities, with obvious swelling and tenderness overlying the small joints of the hands and toes. Groove sign was positive. The joints also displayed restriction of active and passive range of motion. Peripheral pulses were intact and the neurological examination was otherwise unremarkable. Systemic examination was significant for a 3/6 systolic ejection murmur all over the pericardium. Vital signs were then assessed and were normal.

**Figure 1:**
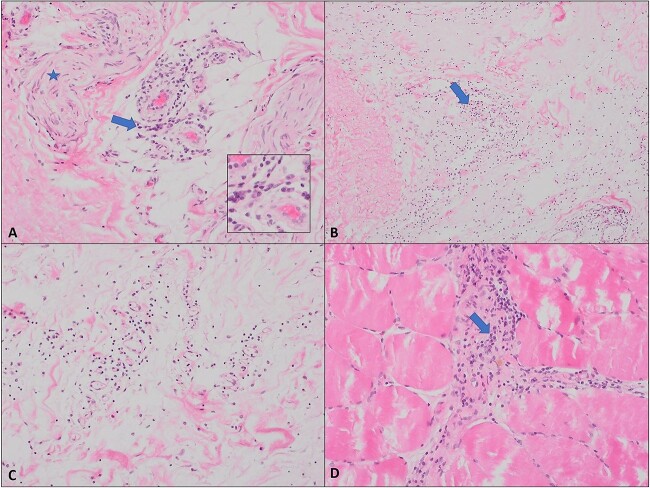
Histopathological examination of the affected skin shows features consistent with eosinophilic fasciitis. (A) Sections show subcutaneous tissue with mild perivascular lymphoplasmacytic inflammation (arrow). The adjacent nerve is marked by a star (The hematoxylin and eosin stain (H&E), 20X). (B and C) The deep soft tissue biopsies demonstrated significant edema with the presence of lymphocytic inflammation (H&E, 10& 20X). (D) Patchy mild lymphoplasmacytic inflammation is noted in the skeletal muscle biopsy (H&E, 20X). No eosinophils are noted in any submitted biopsy.

Further workup revealed leukocytosis (3900/μl) with high eosinophils (20%), and an elevated C-reactive protein (18 mg/l) with negative rheumatoid factor and antinuclear antibody assays (ANA). Other laboratory investigations were normal. Electrocardiogram was unremarkable. Radiological imaging including esophagogastroduodenoscopy, colonoscopy, and computed tomography (CT) Scan of the abdomen and pelvis were unrevealing. Echocardiogram showed mild aortic stenosis. Additionally, a workup for occult malignancy including a bone marrow biopsy and immunohistochemistry was normal. Subsequently, a full-thickness biopsy of the affected skin was done and revealed an inflammatory reaction largely concentrated within the deep fascia and subcutaneous tissue. The inflammatory process was extending deeply into the muscular layer with evidence of mild perivascular chronic inflammation (Fig. 1).

**Figure 2:**
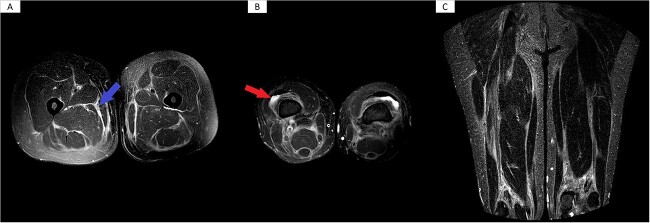
Bilateral MRI of the thighs. (A) MRI showed subcutaneous and deep interstitial edema, as well as mild muscular edema (Arrow on FIgure A). (B) There is evidence of mild bilateral knee joint effusions (Arrow on Figure B). (C) A few left thigh varicose veins are also noted.

Consequently, a magnetic resonance imaging (MRI) of the thighs revealed subcutaneous and deep interstitial edema as well as mild muscular edema and thickening. Moreover, there was evidence of mild effusions in the knee joints bilaterally (Fig. 2). Put together, these findings were suggestive of active fasciitis.

After appropriate consultation, a diagnosis of eosinophilic fasciitis was established. The patient was subsequently started on a tapering dose of prednisolone, as well as calcium and vitamin D supplementation. Five months later, the patient’s skin induration and swelling drastically improved. His laboratory investigations revealed the normalization of inflammatory markers as well as the resolution of eosinophilia.

## DISCUSSION

Eosinophilic fasciitis (EF) is a rare scleroderma-like syndrome causing fibrosis of connective tissues [4]. Interestingly, eosinophilia is a remarkable laboratory finding that can be detected in the early phase of the disease, although it’s less commonly detected in the active and later stages. Moreover, a high creatine kinase, erythrocyte sedimentation rate, hypergammaglobulinemia, and liver enzyme abnormalities could also occur [4]. Clinically, the disorder is characterized by symmetrical soft tissue swelling and skin induration of the distal extremities. This is commonly associated with pain, edema, and tenderness of the affected areas [4]. Eventually, the skin tethers lead to the development of a peau d’ orange appearance [5]. These clinical findings are attributed to eosinophils attacking the subcutaneous layer of the skin leading to induration and thickening [6].

The diagnosis of EF is often challenging and usually requires a full-thickness skin biopsy, which shows lymphocytic and eosinophilic infiltration of the dermis, along with thickened bundles of collagen. Moreover, radiological imaging such as positron emission tomography (PET) CT scan and MRI may play a role in the diagnosis of uncertain cases. For instance, MRI classically shows an increased T2 signal in the subcutaneous and deep fascia of the affected tissue, as well as enhancement of these structures on fat-suppressed T1 images after gadolinium administration [7]. In our case, the skin biopsy results were inconclusive, however, MRI findings strongly correlated with the diagnosis of EF.

The cornerstone therapy for mild to moderately severe EF is oral prednisone. Prednisolone is also commonly used due to its higher bioavailability and potency. A decrease in the induration and pain, along with improvement in the range of motion of the affected joints are expected after initiating therapy and are used as markers for tapering down corticosteroids. Interestingly, most patients remit and terminate therapy within 1–2 years from the initial presentation. Conversely, patients who do not respond to corticosteroid therapy are commonly switched to different immunosuppressive and immunomodulatory medications [8].

Lastly, EF is associated with a wide range of complications including aplastic and hemolytic anemia, idiopathic thrombocytopenic purpura, and myelodysplastic syndrome. Patients who are resistant to steroid therapy commonly develop hematological malignancies years following initial diagnosis [9].
